# *PpERF1b-like* enhances lignin synthesis in pear (*Pyrus pyrifolia*) ‘hard-end’ fruit

**DOI:** 10.3389/fpls.2022.1087388

**Published:** 2022-12-15

**Authors:** Xiaoshan Jin, Chenxia Cheng, Qi Qi, Suping Zhou, Caihong Wang, Yong Zhang, Chao Sun, Yuling Wang, Ruihong Dang, Shaolan Yang

**Affiliations:** ^1^ College of Horticulture, Qingdao Agricultural University, Qingdao, Shandong, China; ^2^ Department of Agricultural and Environmental Sciences, College of Agriculture, Tennessee State University, Nashville, TN, United States; ^3^ Bioengineering College, Aks Vocational and Technical College, Wensu County, Aks, Xinjiang, China

**Keywords:** pear, hard-end, *PpERF1b-like*, ethephon, lignin

## Abstract

The hard-end is a disorder of pear fruit, however, the mechanisms underlying its development remain unknown. In this study, we found that the hard-end fruit contained a higher transcript abundance level of *ethylene-response factor 1b-like* (*PpERF1b-like*) and released more ethylene compared to normal pear. In the ethephon treated normal fruit, flesh tissues accumulated more lignin together with elevated expression of *PpERF1b-like*. Overexpressing *PpERF1b-like* transiently in fruit and stably in callus increased lignin accumulation and the expression of lignin biosynthesis genes; the opposite results were observed in fruit showing repressed expression of *PpERF1b-like*. These results confirmed the role of *PpERF1b-like* in promoting hard-end formation through promoting lignin synthesis. This study provided valuable information for further clarifying the regulation of hard-end formation in pear.

## 1 Introduction

Hard-end fruit is a physiological disorder affecting the marketable quality of pear (*Pyrus pyrifolia)* (Raese, 1993). In recent years, it has been occurring very frequently on pear ‘Whangkeumbae’ (*P. pyrifolia*). The hard-end fruits have protruding calyx with copper-green tough apex (Raese and Drake, 2006). Lu et al. (2015) reported that the hard-end pears contain higher lignin content and more stony cells compared to normal pears, especially a large amount of lignin is deposited in the peel tissue. Lignin deposition on cell wall is critical for plant growth and development (Dixon et al., 2001; Rogers and Campbell, 2004). The secondary cell wall of stony cell is mainly composed of lignin and cellulose (Zhang et al., 2021), and the biosynthesis and deposition of lignin and cellulose in pear flesh are closely related to the generation of stony cells (Cai et al., 2010; Brahem et al., 2017).

The development of hard-end fruit is usually accompanied by lignification (Feng et al., 2021), but the correlation between the two processes remains to be clarified. Ethylene plays a regulatory role in many processes during plant growth and development (Abeles et al., 1992). Studies have shown that ethylene promotes lignification in bamboo shoot (Luo et al., 2021), mung bean (Huang et al., 2013), and loquat (Shan et al., 2008). Ethylene-response factor (ERF) belongs to the *AP2/ERF* (APETALA2/Ethylene-Responsive Factor) transcription factor (TF) superfamily, it plays some roles in providing resistance to biotic and abiotic stresses (Xu et al., 2011). In ethylene treated banana fruit, the expression levels of *MaERF9* and *MaERF11* were increased, which concurs with the involvement of AP2/ERF TFs in ethylene biosynthesis and signal transduction (Xiao et al., 2013).

The first ERF TF was isolated from tobacco (*Nicotiana tabacum)* as a critical gene in ethylene signal transduction pathway of plants (Ohme-Takagi and Shinshi, 1995; Bleecker and Kende, 2000). In recent years, roles of ERF TFs in regulating responses to stress-induced ethylene accumulation have been studied on tomato (Liu et al., 2014; Nakano et al., 2014), kiwifruit (Yin et al., 2012), grape (Licausi et al., 2010), apple (Wang et al., 2007), sweet orange (Xie et al., 2014), and melon (Ma et al., 2015). Additionally, the ERF TFs can also affect the lignification process of plants by influencing lignin biosynthesis. For instance, the *EjAP2-1* which was isolated from loquat (*Eriobotrya japonica*), was reported to inhibit the activity of promoter for the lignin biosynthesis gene *Ej4CL1*, and enhance the activation effect of *EjMYB2* on lignin biosynthesis by interacting with *EJMYB1/2*, thus negatively regulating the lignification of loquat fruit (Zeng et al., 2015). Guo et al. (2016) also reported that in island cotton, *GbERF1-like* up-regulates the expression of genes in lignin biosynthesis pathway, and the increased lignin accumulation helps to improve the resistance of cotton to verticillium wilt.

In our studies, the expression of *PpERF1b-like* TF was found to be significantly up-regulated in hard-end pears by using RNA-Seq analysis (Li et al., 2019) and RT-qPCR verification. The function and mechanism of *PpERF1b-like* TF were investigated using transient overexpression of the gene in normal fruit and transgenic calli derived from flesh tissues of pear ‘Whangkeumbae’. The results presented here will not only help understanding the hand-end formation in pear fruit, but also the process of lignin biosynthesis and its interaction with ethylene release.

## 2 Materials and methods

### 2.1 Plant material

Ten-year-old pear ‘Whangkeumbae’ trees were grown in two orchards located in Laiyang City, Shandong Province, China. Normal fruit were picked in the orchard with low incidence rate (Orchard 1), and the hard-end fruit in the orchard with high incidence rate (Orchard 2). Pears were harvested at 90 and 120 days after anthesis, respectively. Samples were transported to the laboratory within two hours after picking from the trees. Normal fruit of uniform size with no defects stored at 20 °C were used in fruit injection experiments for studying transient overexpression of genes. After removal of seeds or peel, fruit flesh tissues were cut into small pieces (approximately 1 cm^3^), immediately frozen in liquid nitrogen, and stored at -80 °C until analysis.

### 2.2 Ethylene production analysis

The ethylene production rate was measured using fruit harvested at 90 and 120 days after anthesis by using a GC-2010 Plus Gas Chromatograph (Shimadzu, Kyoto, Japan). The hard-end and normal fruit with no visible defects (including damages from wound, disease or insects) were selected and then placed in a closed container for 1 hour. Gas was extracted, and ethylene content was determined according to Yang et al. (2015). Five fruits per sampling point and 3 biological replicates per treatment group were used in ethylene assay.

### 2.3 Quantitative real-time PCR validations

The total RNA was extracted from flesh tissues of pear ‘Whangkeumbae’ using RNA Plant Reagent (TianGen, Shanghai, China) according to the manufacturer’s instructions. The first strand cDNA was synthesized with 1 μg RNA using the HiScript II Q RT SuperMix for qPCR (+ gDNA wiper) (Vazyme, Nanjing, China). The RT-qPCR was performed on a Light Cycler^®^ 480 instrument (Roche, Basel, Switzerland), using a program comprising an initial denaturation at 94 °C for 5 min, followed by 42 cycles of 94 °C for 15 s and 60 °C for 1 min. A negative control without template cDNA for each primer pair was included in each run. For each group of samples, three biological replicates and three technical replicates/sample were included. A pear *Actin* gene (LOC125473976, NCBI database) was used as the house keeping gene (internal reference) to normalize the relative gene transcript abundance levels. The relative gene expression was calculated using the 2^−ΔΔCT^ method (Livak and Schmittgen, 2000). The primers used for the RT-qPCR analysis were designed using NCBI Primer designing tool (https://www.ncbi.nlm.nih.gov/tools/primer-blast/) and are listed in **Table 1**.

**Table 1:** Primers used for RT-qPCR reactions.

Gene name	Primer name	Primer sequence
*PpERF1b-like*	PpERF1b-like-F	5’-GACTCCACTAGACATGGCATAAG-3’
	PpERF1b-like-R	5’-CCTCACATCTCTGTCCACTTTAC-3’
*PpActin*	PpActin-F	5’-CCCAGAAGTGCTCTTCCAAC-3’
	PpActin-R	5’-TTGATCTTCATGCTGCTTGG-3’
*PpCAD1*	PpCAD1-F	5’-GATGTCACAGACCCAAAGGCA-3’
	PpCAD1-R	5’-AGGCGTTCGAGGTTTTCCAT-3’
*PpCAD2*	PpCAD2-F	5’-TTTGGTTGAGAGAGTTGCCCAC-3’
	PpCAD2-R	5’-ATTCGACACCCAAGCTCTTCG-3’
*Pp4CL3*	Pp4CL3-F	5’-ACTCCTACTGCCTCCACAAC -3’
	Pp4CL3-R	5’-GCTGCCTCATCCTTCATTGG -3’

### 2.4 Cloning of *PpERF1b-like*

Isolation of total RNA and synthesis of cDNA followed the same protocol used in RT-qPCR analysis. The PCR reaction was conducted using a program comprising of initial denaturation at 94°C for 5 minutes followed by 35 cycles of 94°C for 30 seconds, 58°C for 30 seconds and 72°C for 1 minute; with a final extension at 72°C for 10 minutes. The PCR primers used to clone *PpERF1b-like* are listed in **Table 2**. The open reading frame (ORF) of *PpERF1b-like* was amplified using the Phanta Max Super-Fidelity DNA Polymerase (Vazyme, Nanjing, China). The PCR products were cloned onto pMD-19-T vector (Takara, Dalian, China).

**Table 2:** Primers used for PCR amplifications of *PpERF1b-like* gene from pear genomic DNA.

Gene name	Primer name	Primer sequence
*PpERF1b-like*	PpERF1b-like-F	5’- ATGCATTGCCACAAACACAC -3’
	PpERF1b-like-R	5’- TCACCAATTAGGAGTGGCATTAG -3’

### 2.5 Vector construction and transient overexpression of *PpERF1b-like* in pear fruit

The full-length cDNA and the antisense fragment of *PpERF1b-like* that were isolated by digestion with restriction enzymes *Xba* I and *Sma* I were ligated onto the expression vector, *Super1300*, driven a 35S promoter. The fusion vectors, *Super1300-sense-PpERF1b-like* and *Super1300-antisense-PpERF1b-like* were introduced into *Agrobacterium tumefaciens* GV3101 using the freeze-thaw method (Weigel and Glazebrook, 2006). The transient overexpression assay of sense-*PpERF1b-like*, antisense-*PpERF1b-like* and empty vector in ‘Whangkeumbae’ normal pear fruit was conducted according to a method described previously (Li et al., 2019). The primers used to construct the expression vectors are listed in **Table 2**.

### 2.6 Subcellular localization of *PpERF1b-like* transcription factor

Subcellular localization of the *PpERF1b-like* TF in onion (*Allium cep*a) cells was conducted using *A. tumefaciens* harboring the *pSuper1300-PpERF1b-like* following the method of Sun et al. (2007) with some modifications. Onion scales were incubated at 28 °C for 24 h in dark. For infection, the onion peels were submerged in *A. tumefaciens* suspension (OD_600_ = 0.6-0.8) and supplemented with acetosyringone (20 mg/L) for 15-20 min. The onion peels were then transferred to 1/2 MS solid medium supplemented with acetosyringone (20 mg/L) followed by incubation at 28 °C for 24 hours in dark. The infected cells producing green fluorescent protein (GFP) were observed and imaged using EVOS™ FL Auto 2 Imaging System (EVOS FL AUTO 2.0, Thermo Fisher, Waltham, America). Positive signals indicate the subcellular localization of *PpERF1b-like* TF.

### 2.7 Ethephon treatment

During harvest season of pear ‘Whangkeumbae’, normal and hard-end fruit were treated with ethephon, and water was used as non-treated control. One half of the fruit was soaked in 400 mg/L ethephon solution and the other half in water, each for 5 minutes. The treated fruits were stored for 3 days before stained with phloroglucinol.

### 2.8 Weisner staining, microscopy and lignin content determination

The Wiesner reaction assay using phloroglucinol-HCl staining was conducted using tissue sections in order to visualize lignified structures (Blanco-Portales et al., 2002). Sections of 2 mm in thickness were cut along the equator of the fruit, and stained with Wiesner reagent for 10 min. Lignified tissues appeared pink or fuchsia in color. Images were captured using a camera (ZXUS 230 HS; Canon, Tokyo, Japan). After staining in the Wiesner reagent for 5 minutes, the thin-sections were observed under a Leica Fluorescence Microscope and imaged using the CCD image acquisition system on the same microscope. The lignin content was measured as previously described by Dyckmans et al. (2002). The stone cell area was counted by Adobe Photoshop 2020.

### 2.9 Transformation of pear calli

The pear calli were induced from the flesh of ‘Nanshui’ (*P. pyrifolia*) of fruitlets in our laboratory, and the pear calli were cultured following the method of Wang et al. (2021). The calli were transformed with *pSuper1300-PpERF1b-like-GFP* and *pSuper1300-GFP* as the empty vector control, using the *Agrobacterium*-mediated genetic transformation method described by Wang et al. (2021) with some modifications. Pear calli were inoculated with *A. tumefaciens* GV3101 harboring the vector constructs after incubation for 20-30 min at 24 °C. After co-culture on plates containing acetosyringone (100 µg/mL) in dark for 3 days, calli were sub-cultured onto selection plates containing cephalosporin (300 mg/L) and hygromycin (10 mg/L) for 25 days. Newly-formed calli were subcultured to fresh selection media monthly until tissues with no sign of brown and died cells were obtained. Tissue culture was conducted at 24 °C in a growth chamber.

### 2.10 Statistical analyses

Data were analyzed using Microsoft Excel software. Significant differences between sample groups were calculated using GraphPad Prism 8 and data were graphed in the same program.

## 3 Result

### 3.1 Ethylene production rate of pear ‘Whangkeumbae’ before harvest

**Figure 1:**
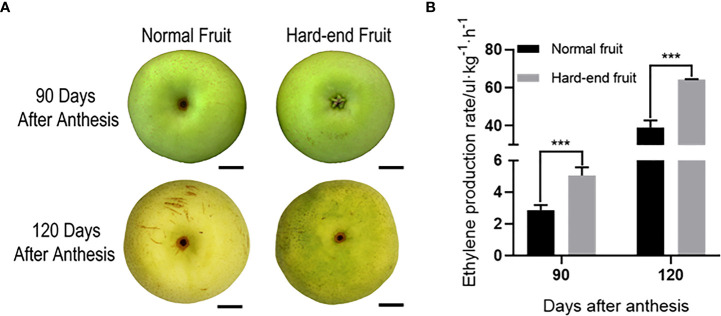
Ethylene production rate of ‘Whangkeumbae’ at 90 and 120 days after anthesis. **(A)** Phenotypes of normal and hard-end fruit of pear ‘Whangkeumbae’ at 90 and 120 days after anthesis. Scale bars = 150μm. **(B)** Ethylene production rate of normal and hard-end fruit at 90 and 120 days after anthesis. Data are given as mean ± SD (n = 4). The asterisk represents a significant difference (***p <0.001; Student’s *t-test*).

Ethylene production rate was measured for normal and hard-end fruit with obvious phenotype at 90 and 120 days after anthesis (**Figure 1A**), respectively. The ethylene production rate of hard-end fruit was higher than the normal fruit on both sampling dates (**Figure 1B**). The results showed that ethylene production was positively correlated with the occurrence of hard-end pear fruit.

### 3.2 Screening of *PpERF1b-like*

**Figure 2:**
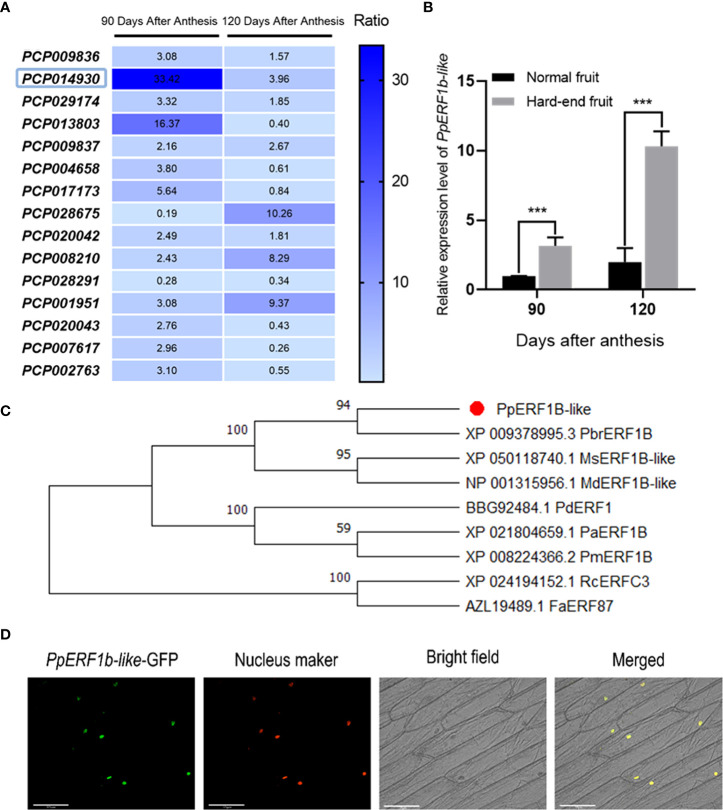
Transcriptome sequencing and subcellular localization of *PpERF1b-like*. **(A)** Screening of *PpERF1b-like* by transcriptome sequencing. **(B)** The relative expression level of *PpERF1b-like* in normal and hard-end fruit of pear ‘Whangkeumbae’ at 90 and 120 days after anthesis. Data are given as mean ± SD (n = 4). The asterisk represents a significant difference (***p <0.001; Student’s *t-test*). **(C)** Phylogenetic analysis of *PpERF1b-like*. *PpERF1b-like* protein sequences (indicated by red figure) were aligned with ERFs from *P. bretschneideri Rehd* (Pbr), *Malus sylvestris* (Ms), *M. domestica* (Md), *P. dulcis* (Pd), *P. avium* (Pa), *P. mume* (Pm) and *Rosa chinensis* (Rc). The phylogenetic tree was constructed using the neighbor-joining method with 1000 bootstrap replications in MEGA 11 software. Sequences were obtained from the National Center for Biotechnology Information nucleotide database (http://www.ncbi.nlm.nih.gov/nucleotide/). Bootstrap values are indicated above the branches. **(D)** Subcellular localization of *PpERF1b-like*-green fluorescent protein (GFP) fusion protein was determined by infecting onion epidermal cells and imaged under a EVOS FL Auto 2 imaging system. Scale bars = 125μm.

*PpERF1b-like* was identified as one of the different expressed genes (DEGs) between hard-end and normal fruit harvested on 90 and 120 days after anthesis. The raw RNA seq sequences were deposited in NCBI database (NCBI SRA Accession: SRP063385). In this study, the transcript level of *PpERF1b-like* in pear ‘Whangkeumbae’ was higher in hard-end fruit harvested on the 90 days after anthesis (**Figure 2A**). Moreover, results from RT-qPCR analysis showed that the relative transcript abundance level of *PpERF1b-like* in hard-end fruit was significantly higher than that in normal fruit from the two harvests (**Figure 2B**). These data indicate a positive correlation between the expression of *PpERF1b-like* and the occurrence of hard-end fruit. A phylogenetic tree was constructed using the amino acid sequence of *PpERF1b-lik*e and other ERFs from a variety of plant species. The results showed that *PpERF1b-like* protein has the closest relationship with *PbrERF1b* protein from *P. bretschneideri Rehd* (**Figure 2C**). The analysis for subcellular localization confirmed that the *PpERF1b-like-GFP* fusion protein was localized in the nuclei of onion epidermal cells, indicating that the *PpERF1b-like* is a nucleus protein (**Figure 2D**).

### 3.3 Effect of ethephon treatment on lignification of pears

**Figure 3:**
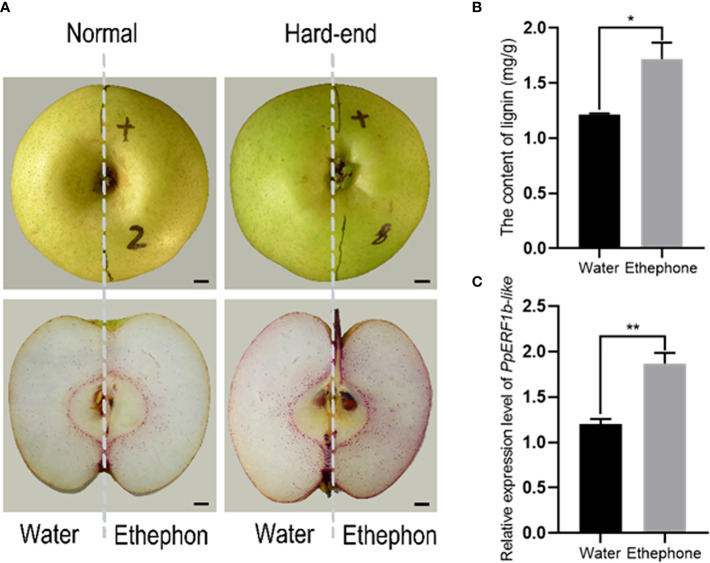
Ethephon promoted lignin accumulation and induced the expression of *PpERF1b-like*. **(A)** Half of the fruit was treated with water and the other half with ethephon. Scale bars = 100μm. **(B)**, The content of lignin. **(C)** The relative expression level of *PpERF1b-like*. Data are given as mean ± SD (n = 3). The asterisk represents a significant difference (*p <0.05; **p <0.01) using Student’s *t-test*.

The effect of ethephon treatment on fruit lignification was further analyzed using normal and hard-end fruits treated with 400 mg/L ethephon. Based on the intensity of lignin staining, both normal and hard-end fruit accumulated more lignin after ethephon treatments compared to the control group (**Figure 3A**). The lignin content of ethephon treated fruits was significantly higher than that of the water-treated control samples (**Figure 3B**). The relative expression level of *PpERF1b-like* showed the same pattern with higher abundance of transcripts in ethephon-treated compared to the non-treated groups (**Figure 3C**). These results indicate that accumulation of lignin and the expression of *PpERF1b-like* were enhanced by ethephon treatment of pears.

### 3.4 Transient expression of *PpERF1b-like* in pears

**Figure 4:**
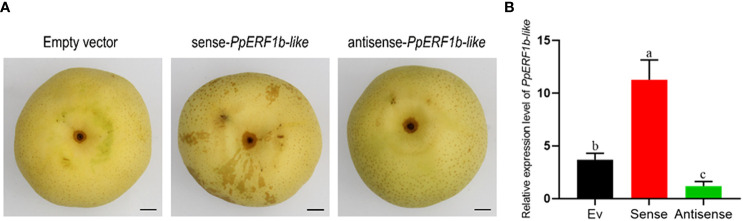
The relative expression level of *PpERF1b-like* in pear fruit surrounding the site of infiltration.in transient overexpression fruit. **(A)** Phenotype of transient overexpression of fruit. Scale bars = 100μm. **(B)** The relative expression level of *PpERF1b-like* in pear fruit surrounding the site of infiltration. Data are given as mean ± SD (n = 4). a-c, the different letters indicate significant differences between groups (*p <*0.05; Duncan’s multiple-range test). Ev: Empty vector, Sense: Sense-*PpERF1b-like*, Antisense: Antisense-*PpERF1b-like*.

On the fruit, the sites inoculated with the *PpERF1b-like* overexpression vector appeared to be more lignified (**Figure 4A**). The expression of *PpERF1b-like* was analyzed in fruit tissues surrounding the injection site. The relative expression level of *PpERF1b-like* in fruit injected with the sense-*PpERF1b-like* vector was significantly higher than the control (inoculated with empty vector), whereas it was significantly repressed by inoculation of the antisense-*PpERF1b-like* vector (**Figure 4B**).

### 3.5 Transient expression of *PpERF1b-like* promoted lignin accumulation and the expression of lignin-related genes in normal fruit

**Figure 5:**
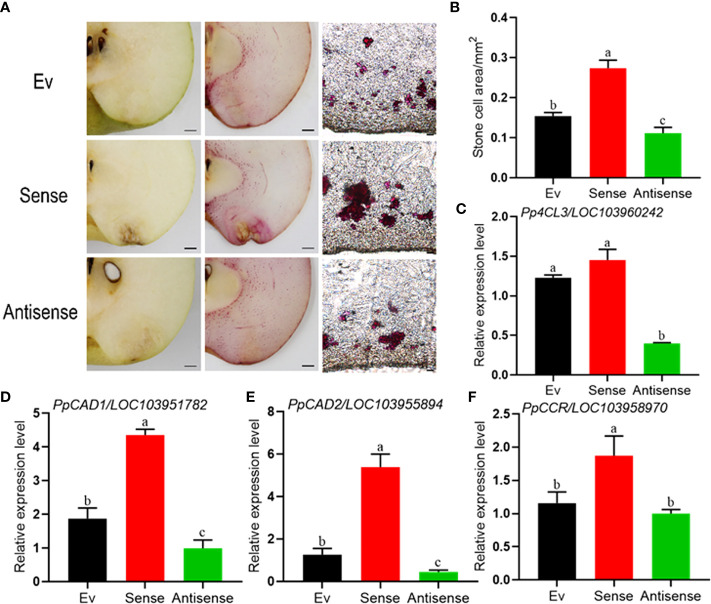
Transient overexpression of sense-*PpERF1b-like* promotes lignin synthesis and the relative expression levels of lignin synthesis related genes in pear fruit surrounding the site of infiltration. **(A)** Lignin staining and frozen section staining in the injection site of transient overexpression fruit. Ev: Empty vector, Sense: sense-*PpERF1b-like*, Antisense: antisense-*PpERF1b-like*. Scale bars = 100μm. **(B)** The area of stone cells in the injection site. Data are given as mean ± SD (n=4). Bars with different letters indicate significant differences between groups (*p <*0.05; Student’s *t-test*). **(C–F)** The relative expression levels of lignin synthesis related genes (*Pp4CL3*, *PpCAD1*, *PpCAD2*, *PpCCR*) in transient overexpression fruit. Data are given as mean ± SD (n=4). a-c, the different letters indicate significant differences between groups (*p <*0.05; Duncan’s multiple-range test). Ev: Empty vector, Sense: sense-*PpERF1b-like*, Antisense: antisense-*PpERF1b-like*.

In order to clarify the function of *PpERF1b-like* in regulating lignin biosynthesis in pear, the thin-sections of fruit inoculated with the gene constructs were stained in Wiesner reagent. As shown in **Figure 5A**, the lignin stain of fruit with transient overexpression of *PpERF1b-like* gene was much deeper than the transient overexpression assay of antisense-*PpERF1b-like* and empty vector. The frozen tissue section prepared using the inoculated fruit also showed that the number of stony cells in the injection site of *PpERF1b-like* gene increased significantly, whereas the injection site with antisense-*PpERF1b-like* contained a smaller number of stony cells (**Figure 5A**). Furthermore, the fruit injection site with *PpERF1b-like* gene has the largest area occupied with stony cells (**Figure 5B**). These results demonstrated that *PpERF1b-like* has a positive regulatory effect on fruit lignification of ‘Whangkeumbae’ pear. The expression of lignin biosynthesis related genes was analyzed in tissues collected around the injection site. As shown in **Figures 5C–F**, the relative expression levels of four genes related to lignin biosynthesis, *Pp4CL3* (**Figure 5C**), *PpCAD1* (**Figure 5D**), *PpCAD2* (**Figure 5E**) and *PpCCR* (**Figure 5F**), were significantly higher in fruit showing transient overexpression of *PpERF1b-like*, compared to the control group. Conversely, the expression of these genes was repressed in fruit with overexpression of antisense-*PpERF1b-like*. When combining all the data from lignin staining and changes in the expression of genes affecting lignin biosynthesis, it can be concluded that the *PpERF1b-like* gene has a positive regulatory function during lignin synthesis which affects the occurrence of hard-end fruit.

### 3.6 Agrobacterium tumefaciens mediated genetic transformation of calli derived from pear flesh tissues

**Figure 6:**
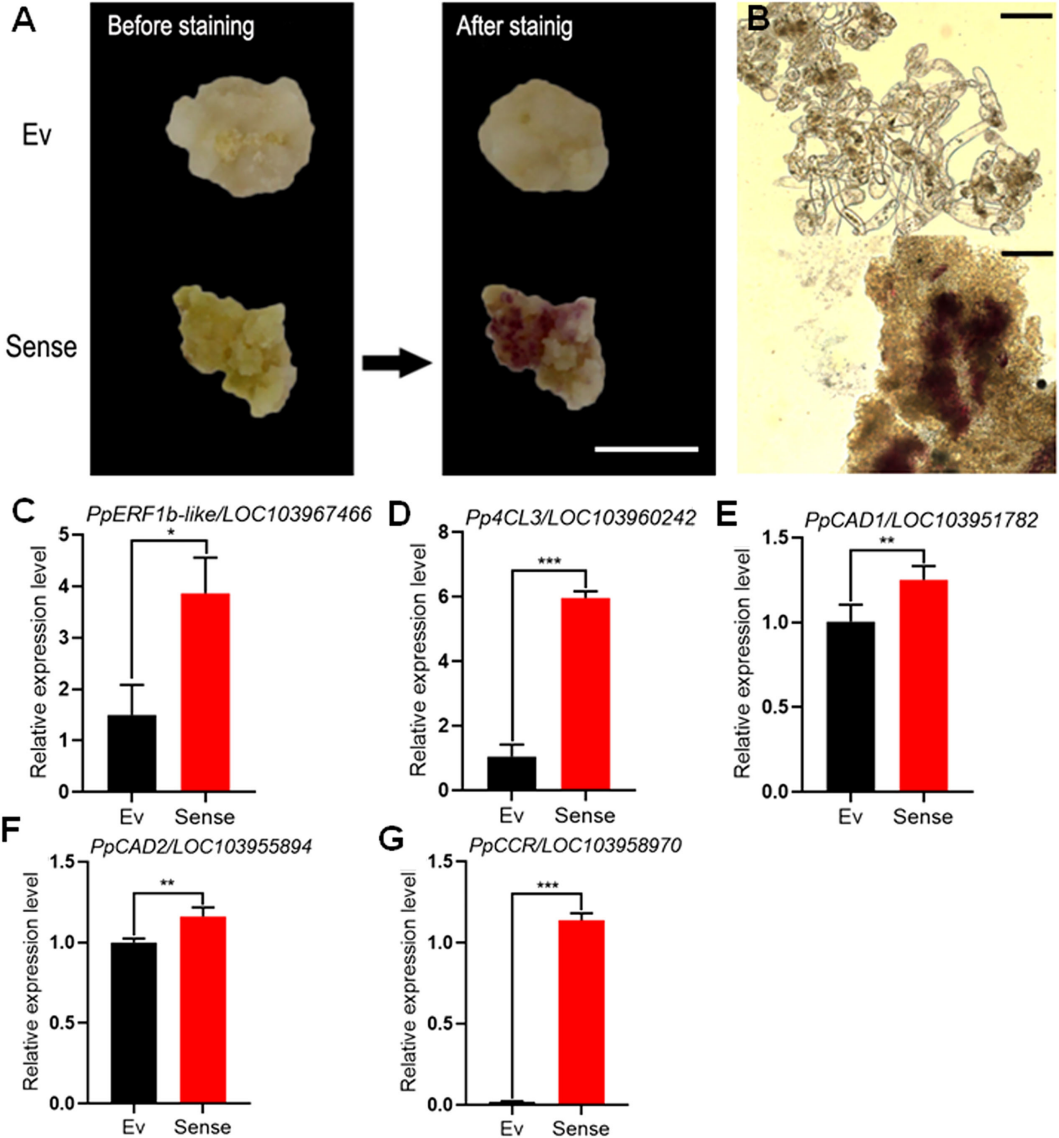
Genetic transformation of callus. **(A)** Whole tissue lignin staining. Scale bar = 100μm. Ev: Empty vector, Sense: sense-*PpERF1b-like*. **(B)** Lignin staining of thin-sections of pear. Scale bars = 50μm. **(C)** The relative expression level of *PpERF1b-like* in transgenic callus. Data are given as mean ± SD (n = 4). The asterisk represents a significant difference (*p < 0.05; **p < 0.01; Student’s *t-test*). **(D–G)** The relative expression levels of lignin biosynthesis related genes (*Pp4CL3*, *PpCAD1*, *PpCAD2*, *PpCCR*) in transgenic calli. Data are given as mean ± SD (n = 4). The asterisk represents a significant difference (*p < 0.05; **p < 0.01; ***p < 0.001; Student’s *t-test*). Ev: Empty vector, Sense: sense-*PpERF1b-like*.

Transgenic calli with stable overexpression of *PpERF1b-like* were stained with Wiesner reagents (**Figures 6A, B**). Calli infected with *PpERF1b-like* turned red which corresponds to high lignin content, whereas no such changes were shown in calli inoculated with the control (empty vector). The relative expression level of *PpERF1b-like* in *PpERF1b-like*-overexpressing pear calli was significantly higher than the control transformed with the empty vector (**Figure 6C**). The relative expression levels of lignin biosynthesis related genes, *Pp4CL3* (**Figure 6D**), *PpCAD1* (**Figure 6E**), *PpCAD2* (**Figure 6F**) and *PpCCR* (**Figure 6G**), were also significantly higher in *PpERF1b-like*-overexpressing calli than those from the control group (**Figures 6D–G**). These results further validated the role of *PpERF1b-like* TF in enhancing lignin biosynthesis.

## 4 Discussion

*APETALA2*/Ethylene Response Factor (AP2/ERF) TFs comprise one of the largest TF families in plants, and they are involved in a variety of signal transduction and growth regulation (Gu et al., 2017). The ERF subfamily is primarily located downstream of ethylene signal transduction and is involved in many biochemical pathways, such as metabolic regulation (Yu et al., 2012), responses to biotic and abiotic stress factors (Yao et al., 2017), hormone signal transduction pathways in plants (Müller and Munné-Bosch, 2015) and plant development (Banno et al., 2001). This study provided experimental evidences for the function of an ethylene response factor, *PpERF1b-like*, isolated from pear ‘Whangkeumbae’. In fruit harvested at 90 and 120 days after anthesis, the ethylene production rate and the relative expression level of *PpERF1b-like* in hard-end fruit were both higher than those in normal fruit. It can thus postulate that the expression level of *PpERF1b-like* is positively related with the physiological disorder in pear.

Lignin is a group of aromatic polymers formed by oxidative coupling of 4-hydroxyphenylpropane through phenylpropane metabolic pathway (Boerjan et al., 2003; Ralph et al., 2004). When plants are challenged with stress conditions, lignin deposition on the secondary cell wall leads to wall thickening which increases the hardness of fruit (Tao et al., 2009; Vanholme et al., 2010). This study shows that ethephon treatment promoted the accumulation of lignin, and the number of stone cells in the treated fruits was also increased as well as the expression of ethylene response factor *PpERF1b-like*. In a study using ethephon treated mung bean roots, it induced expression of peroxidase (POD) which is a key gene catalyzing lignin formation, and more extensive lignification of stems (Huang et al., 2013). As a response factor of ethylene, *PpERF1b-like* may also participate in the regulation of lignin biosynthesis similarly, and eventually affects the occurrence of hard-end fruit in pear.

Based on results from lignin staining, pear flesh tissues became more lignified when they were injected with the sense-*PpERF1b-like-*overexpressing vector, and concurrently, the number of stony cells was also increased, compared to the tissues injected with empty vector. However, there was no significant change in the degree of lignification in tissues injected with antisense-*PpERF1b-like* in spite of fewer stony cells. In Wiesner staining, pear calli infected with *PpERF1b-like* turned red, but this reaction was not obvious in pear calli infected with empty vector. Our results are consistent with a previous study on pear ‘Whangkeumbae’ where lignin staining, lignin content and the number of stony cells in hard-end fruit were all significantly higher than normal fruit (Lu et al., 2015). Taken together, these results indicate that *PpERF1b-like* should positively regulate the synthesis of lignin.

According to a study on *Isatis indigotica*, the AP2/ERF TFs positively regulate lignin biosynthesis by activating salicylic acid signal transduction and lignin pathway genes (Ma et al., 2017). Lasserre et al. (2008) also found that the expression level of *AtERF38* gene was positively correlated with the thickening of secondary wall in seeds and this process is intertwined with lignin biosynthesis. In this study, overexpressing sense-*PpERF1b-like* transiently in pear ‘Whangkeumbae’ fruit or stably in calli both led to an elevated levels of expression of *PpERF1b-like* and lignin biosynthesis related genes. On the contrary, expression of these genes were repressed in the fruit injected with antisense-*PpERF1b-like*. Future studies will investigate if the *PpERF1b-like* gene directly or indirectly regulate lignin biosynthesis genes, as well as its connection to the formation of hard-end pears.

## 5 Conclusion

This study reports that the relative expression level of *PpERF1b-like* was positively correlated with higher lignin content in hard-end pears, indicating that *PpERF1b-like* is a key gene affecting fruit lignification. Results from transient expression of *PpERF1b-like* in normal fruit of pear ‘Whangkeumbae’ and in callus tissue provided experimental evidences supporting the role of *PpERF1b-like* in regulating the expression of lignin-related genes, and affecting the occurrence of fruit calyx end hardening.

## Author’s note

We confirm that the manuscript has been read and approved by all named authors and that there are no other persons who satisfied the criteria for authorship but are not listed. We further confirm that the order of authors listed in the manuscript has been approved by all of us.

## Data availability statement

Publicly available datasets were analyzed in this study. This data can be found here: NCBI SRA Accession: SRP063385.

## Author contributions

Study design: CC, RD, and SY. Data collection: CC, YZ, CS, and QQ. Data analysis: XJ, CC, CW, and SY. Data interpretation: CC. Software: XJ, RD, and YZ. Supervision: CW, RD, and SY. Visualization: XJ, QQ, and RD. Writing—original draft: XJ and SZ. Writing—review and editing: XJ, CC, SZ, RD and SY. All authors contributed to the article and approved the submitted version.
